# Use of Cyanobacterial Luminescent Bioreporters to Report on the Environmental Impact of Metallic Nanoparticles

**DOI:** 10.3390/s19163597

**Published:** 2019-08-19

**Authors:** Jara Hurtado-Gallego, Francisco Leganés, Roberto Rosal, Francisca Fernández-Piñas

**Affiliations:** 1 Departamento de Biología, Facultad de ciencias, Universidad Autónoma de Madrid, 28029 Madrid, Spain; 2 Departamento de Ingeniería Química, Universidad de Alcalá, 28871 Alcalá de Henares, Spain

**Keywords:** cyanobacterial bioreporters, toxicity, oxidative stress, metallic nanoparticles, released free-ion

## Abstract

Due to their ecological relevance, low cost, and easy maintenance, cyanobacteria have been used for bioreporter development. In this study, a battery of cyanobacterial bioreporters has been used to assess the ecotoxicity of four highly used metallic nanoparticles (NPs). The toxicity of these NPs was tested using the bioreporter *Nostoc* CPB4337 (*Anabaena* CPB4337). As oxidative stress is a primary toxic mechanism of metallic NPs, cyanobacterial reactive oxygen species (ROS)-detecting bioreporters were used. Metallic NPs release metal ions, which contribute to their toxic effect and the formation of ROS, so a metal-detecting bioreporter was also used to detect the bioavailable metals. The results confirm that ROS production by NPs was due to the NPs per se and not by released free-ions, which in fact were almost undetectable. Although the metal-detecting bioreporter could not detect the dissolved metal ions, it was able to detect the metallic NPs themselves, indicating that this bioreporter may be useful to detect them in the environment. ROS production varied depending on the growth medium or environmental matrices conditions and on the NP type. This work demonstrated the different levels of ROS production by metallic NPs and the importance of nanotoxicology studies in real matrices.

## 1. Introduction

Heavy metals are naturally occurring elements in the earth but anthropogenic activities such as mining and smelting operations, industrial production and use, and domestic and agricultural use of metals and metal-containing compounds has dramatically increased their presence in the natural environment [1]. Some metals such as cobalt (Co), copper (Cu), iron (Fe), magnesium (Mg), manganese (Mn), molybdenum (Mo), nickel (Ni), and zinc (Zn) are essential nutrients that are required for various biochemical and physiological functions [2], however, high concentrations of these metals can be toxic to the organisms [3]. Other metals such as silver (Ag), cadmium (Cd), mercury (Hg), or titanium (TiO_2_) which are considered as non-essential metals are toxic for most organisms at very low concentrations [3,4].

Nowadays, diverse heavy metals have been used in the development of nanoparticles (NPs) [5]. Metallic NPs have been progressively involved in the development of a number of new applications in the most diverse fields. These fields include nanomedicine, the production of consumer goods and materials for environmental remediation. Their rapid expansion and expected release are still controversial and raise serious concerns about their impact on human health and on the environment. In developed countries, the marketing of new technological products is expected to meet stringent environmental responsibility criteria. This requires conducting bioassays with organisms representing all environmental compartments, and to take into account all metallic NPs properties that make their interaction with living cells distinct from bulk conventional materials. These properties include their small size and their high surface/volume ratio, which lead to an enhanced surface reactivity. The data available on the mechanism of action of metallic NPs on living organisms are scarce and sometimes contradictory, this being particularly true for free-living microorganisms.

Like other pollutants, metallic NPs induce reactive oxygen species (ROS) formation in a large variety of organisms [6,7,8]. These ROS are natural byproducts of aerobic cellular oxidative metabolism and they have essential roles in the cell survival pathways [9,10]. ROS include free radicals such as superoxide (O_2_^−^) or hydroxyl (HO^.^) radicals with a short lifespan and nonradicals such as such as hydrogen peroxide (H_2_O_2_) or organic peroxides (ROOH) which are more stable molecules [6,11]. In normal conditions, ROS production and their detoxification is carried out by specific enzymes such as superoxide dismutases, peroxiredoxins or catalases. However, when ROS production increases, an imbalance occurs. This imbalance is known as oxidative stress.

As previously mentioned, the wide use of NPs makes it necessary to study their environmental impact. Although more mechanistic studies are necessary, there are a large variety of ecotoxicological studies that confirm the production of ROS by NPs [12,13]. Furthermore, metallic NPs may release metal ions that contribute to their toxic effect and also to ROS formation [14]. ROS production by NPs are studied by diverse methods from detection via fluorescence measurement of oxidized fluorescent dyes to indirect methods such as analysis of gene expression and determination of antioxidant inhibition of NP cytotoxic effects as summarized by Zuberek et al. (2018) [15]. However, the current assessment of NP toxicity in real waters is limited due to a lack of suitable methodologies [16,17,18]. An alternative methodology suitable in ecotoxicology is the use of bacterial bioreporters [19,20]. Furthermore, due to their easy maintenance, rapid growth and portable devices light-emitting bioreporters give an advantage for screening in nanotoxicology [21,22]. Cyanobacteria are gram-negative photosynthetic bacteria ubiquitous in many environments. Besides, they are primary producers so any deleterious effect on them will affect the rest of the trophic chain so they are representative of the health of the environment they live in [23,24]. Due to their easy maintenance and the broad knowledge about their molecular biology [25], cyanobacteria have been used to construct by genetic engineering a wide variety of bioreporters, demonstrating a useful tool for environmental assessment [26,27].

The goal of this study is toxicity assessment of selected metallic NPs using a battery of cyanobacterial bioreporters highlighting ROS formation as well as released (dissolved) free-ions. With this purpose, the following recombinant bioluminescent cyanobacterial strains were used: general toxicity bioreporter *Nostoc* CPB4337 [28] (formerly denoted as *Anabaena* CPB4337); ROS-detecting *Nostoc* sp. PCC7120 pBG2154, *Nostoc* sp. PCC7120 pBG2165 [29] and *Nostoc* sp. PCC7120 pBG2173 [30] which detect specifically superoxide anion and *Nostoc* sp. PCC7120 pB2172 [30] which detects both superoxide anion and H_2_O_2_. *Synechococcus* sp. PCC7942 pBG2120 which reports on bioavailable metal free-ions was also used [31]. All these bioreporters harbour *luxCDABE* reporter genes from the terrestrial bacterium *Photorhabdus luminescens* fused to promoters of genes responsive to toxicity, oxidative stress, and heavy metals, respectively. Furthermore, due to the importance of testing the bioreporter response in real environmental samples, the ROS-detecting bioreporters have been tested in river and wastewater samples spiked with the metallic NPs.

## 2. Materials and Methods

### 2.1. Biological Materials and Culture Conditions

All the bacterial strains used in this study and their culture conditions are summarized in Table 1. Briefly the bioluminescent transformed strains based on *Nostoc* sp. PCC7120 which detect ROS [29,30] were grown in AA/8+N culture medium at 28 °C with continuous illumination, at 60 µmol photons m²sˉ¹ on a rotatory shaker in 250 mL Erlenmeyer flasks. Culture medium was supplemented with spectinomycin (Sp) (2 µg/mL). Transgenic bioluminescent *Synechococcus elongatus* sp. PCC7942 pBG2120 [31] was grown in BG11 medium buffered with 2 mM MOPS and pH 7.5. The culture medium was supplemented with 3.75 µg/mL of chloramphenicol (Cm). *Nostoc* sp. CPB4337 was grown in AA/8+N medium supplemented with neomycin (Nm) (3.2 µg/mL). The culture conditions for the latter strains were the same as those of *Nostoc* sp. PCC7120 strains.

### 2.2. Chemicals

Metallic NPs used for the experiments were ZnONPs, Cu_2_ONPs (Sigma-Aldrich), TiO_2_NPs and AgNPs (Plasmachem) (denoted as ZnNPs, CuNPs, TiNPs and AgNPs, respectively); particle sizes were ˂50 nm, ˂50 nm, 4–8 nm, and 10 nm, respectively.

The stock solutions were prepared with distilled water sonicated in an ultrasonicator bath for 15 min and further stored at 4 °C in darkness.

### 2.3. Characterization of NPs

Hydrodynamic diameter and ζ-potential of the NPs suspensions in the different assay conditions were measured by Dynamic light scattering (DLS) and electrophoretic light scattering respectively using a Zetasizer Nano ZS particle size analyzer from Malvern Instruments Ltd. Measurements were essentially as described previously by Gonzalo et al. (2014) [33].

### 2.4. Bioluminescence Assays

Firstly, toxicity of metallic NPs was assessed by the use of the toxicity cyanobacterial bioreporter *Nostoc* sp. CPB4337; a general toxicity recombinant bioluminescent bioreporter used previously for testing metal toxicity [4,28], emerging pollutants [34,35,36] and nanoparticles toxicity [37,38,39]. Toxicity data were used to calculate IC_50_ (the half maximal inhibitory concentration) values (see statistical analysis below).

The potential generation of ROS by the metallic NPs was tested by using ROS-detecting *Nostoc* sp. PCC7120 bioreporters (Table 1). These bioreporters have been previously used to detect ROS formation caused by the herbicide methyl viologen (MV or paraquat), H_2_O_2_, and the emerging pollutant triclosan [29,30].

Metallic NPs have been described to release metal ions [40,41], so that their toxicity may be due at least in part to the released free-ion. In order to detect potential released free-ions, the metallic NPs suspensions were treated by centrifugal ultrafiltration through a membrane (Sartorius AG, Goettingen, Germany) with a nominal molecular weight limit of 50 kDa (Vivaspin 6). Suspensions were centrifuged for 15 min at 4000 rpm (Allegra X-12 Series, Beckman Coulter, Brea, CA, USA). The filtrates collected were analyzed by total reflection X-ray fluorescence (TXRF) and used for the experiments concerning free released ions from NPs. Besides TXRF, *Synechococcus* sp. PCC7942, a heavy metal-detecting bioluminescent bioreporter [31] was used to detect the bioavailability of these released ions.

The bioluminescence assays were performed with the NPs indicated above in the following concentration ranges (resulting from preliminary experiments): AgNP 0.12–5 mg/L, CuNP 1.6–25 mg/L, TiNP 15–75 mg/L and ZnNP 0.06–1 mg/L.

Before the exposure experiments, all the cyanobacterial bioreporters were grown until reaching the mid-log phase [based on their optical density (OD)] (OD_750nm_ = 0.5 − 0.6) because this growth stage was found to be the optimal for bioluminescent assays [29] and were washed twice in each specific medium (AA/8 + N or BG11 see Table 1). For standardization purposes, cells were resuspended in fresh specific medium at a final OD_750nm_ = 0.5 [28]. Exposure experiments were performed in transparent 24 well microplates in 1.5 mL final volume [31]. Metallic NPs were then added to the wells to obtain the desired final concentrations.

Plates were incubated at 28 °C in light (60 μmol m^2^s^−1^) on a rotary shaker for 24 h. For the luminescence measurements, 100 μL of cell suspensions were transferred to an opaque 96-well microliter plate and luminescence was recorded every 1 min for 10 min in a Centro LB960 luminometer (Berthold Technologies GmbH and Co.KG, Bald Wilbad, Germany) and the maximum value recorded was taken.

### 2.5. Spiking Experiments: Performance of the ROS-Detecting Bioreporters in Environmental Matrices Artificially Contaminated with Metallic NPs

In order to evaluate the response of ROS-detecting bioreporters in natural matrices after the exposure to metallic NPs, two types of natural water were used. Both natural waters have been previously characterized and described. Briefly, pristine water (Glx1) coming from near the head waters of Guadalix River [42] and polluted water coming from a wastewater treatment plant (WWTP) [43,44] were used. For the spiking experiments, the same conditions of bioluminescence assays previously described were carried out but using the natural waters; cells were resuspended in the specific natural water at a final OD_750nm_ = 0.5. Exposure experiments were performed in transparent 24 well microplates in 1.5 mL final volume. Metallic NPs were then added to the wells to obtain the desired final concentrations. The plates incubation and the bioluminescent measurements were the same that in the “bioluminescence bioassays” section.

### 2.6. Statistical Analysis

All data were obtained from a minimum of three independent experiments with three replicates for each assay situation. The toxicity results using *Nostoc* CPB4337 were expressed as a percentage of bioluminescence inhibition. This bioassay is based on the inhibition of constitutive luminescence caused by the presence of a toxic substance [28]. Toxicity was expressed as inhibitory concentration IC_50_ which is the toxic compound concentration exerting 50% bioluminescence inhibition. To calculate the IC_50_ values, dose-response curves were fitted by non-linear parametric functions with the R“*drc*” analysis package (Ritz and Streibig, 2005) [45] (R for windows, 3.0.2versionDevelopment Core Team, Vienna, Austria). Best-fit models were selected by using the “model select” function provided in the *drc* package according to the maximum likelihood and the Akaike’s information criterion [45].

For inducible bioreporters (ROS-detecting *Nostoc* sp. bioreporters and *Synechococcus elongatus* sp. PCC7942 pBG2120), data are expressed as bioluminescence induction factors (BIFs) calculated by dividing the mean luminescence signal of a treated sample by the mean luminescence signal of the untreated sample. All tests of statistically significant differences between data sets were performed using the Student’s t test and one-way analysis of variance (ANOVA), both of which were computed using R analysis package (R for windows, 3.0.2 copyright© The Foundation for Statistical Computing, Vienna, Austria).

## 3. Results

### 3.1. Metallic NPs Physicochemical Characterization

The properties of the metallic NPs in pure water, AA/8+ N and BG11 growth medium and in natural matrices Glx1 and WWTP are listed in Table 2. The hydrodynamic diameter of metallic NPs was measured by DLS and the net charges by *ζ*-potential at the concentration corresponding to calculated IC_50_ (see below). In AA/8+N medium, the particle *ζ*-potential value was essentially coincident with that of the medium without NPs maintaining colloidal stability, while in BG11 medium the *ζ*-potential values were lower with respect to the medium without NPs indicating less colloidal stability. Although these differences between media, all the NPs presented a negative charge in all the tested matrices (Table 2). Interestingly, the *ζ*-potential values in pure water were similar than those in Glx1 natural water. However, these *ζ*-potential values changed slightly in wastewater in which the *ζ*-potential was essentially coincident with that of wastewater particles probably due to heteroaggregation with natural colloids as previously observed by Martín-de-Lucía et al. (2017) [39]. Accordingly, the suspensions of NPs in WWTP led to colloids with a size distribution in the hundreds of nanometer range (Table 2). As can be observed in Table 2, the NPs presented different characteristics in the diverse matrices. AgNPs tended to form aggregates of different sizes in all the matrices, showing at least two sizes peaks which correspond to different aggregation levels, probably in a dynamic equilibrium with the larger ones. The rest of the NPs did not show different size peaks.

### 3.2. Response of Nostoc sp. CPB4337 to Metallic NPs: Toxicity

*Nostoc* sp. CPB4337 was used for evaluating the toxicity of the metallic NPs. Table 3 shows the inhibitory concentrations (ICs) of metallic NPs that inhibit 10%, 50% and 90% the bioluminescence of the bioreporter at 1, 6 and 24 h of exposure to the NPs. As an example, the dose–response profiles of metallic NPs for *Nostoc* sp. CPB4337 after 24 h of exposure (from where the information in Table 3 was obtained) can be found in Figure S1. As can be observed in the table, except for AgNPs, after 1 h of exposure, the IC50 values were remarkably higher than those after 24 h of exposure as toxicity increases over time, in fact, for TiNPs no IC value was found for the range of tested concentrations (0.01 mg/L to 100 mg/L) which is in agreement with the lower toxicity of TiNPs; after 6 h of exposure, a similar trend is observed with the exception of AgNPs. The fact that AgNPs seem to be less toxic at 24 h of exposure than at shorter times of exposure probably has to do with colloidal stability issues and the formation of aggregates of different sizes (see Table 2) as previously reported by our group (Gonzalo et al. 2014). After 24 h of exposure, all metallic NPs exerted clear toxicity towards *Nostoc* sp. CPB4337; the IC_10_, IC_50_ and IC_90_ values of ZnNPs were lower than those of the other NPs, indicating that ZnNPs were more toxic towards the cyanobacterium followed by AgNPs, CuNPs and TiNPs which were the least toxic.

### 3.3. Response of Synechococcus Elongatus sp. PCC7942 pBG2120 to Released Free-Ions from Metallic NPs

**Figure 1:**
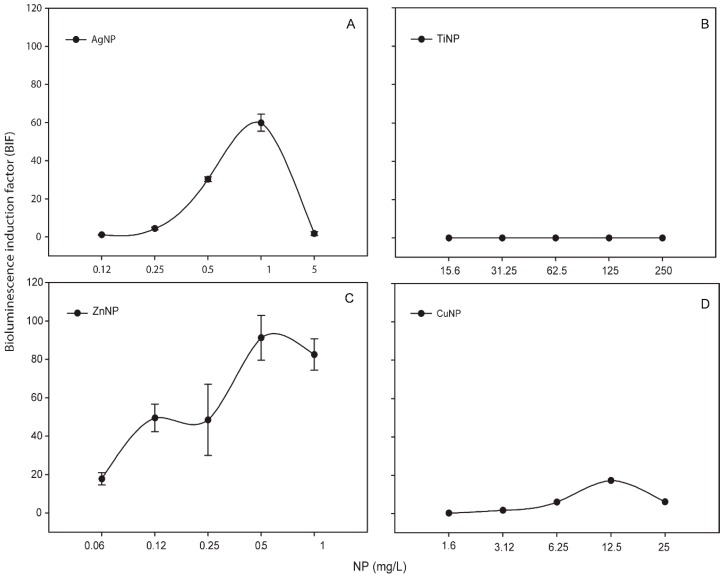
Bioluminescence response of *Synechococcus elongatus* sp. PCC7942 pBG2120 exposed to metallic NPs after 4 h of exposure. (**A**) *Synechococcus elongatus* sp. PCC7942 pBG2120 exposed to AgNPs; (**B**) *Synechococcus elongatus* sp. PCC7942 pBG2120 exposed to TiNPs; (**C**) *Synechococcus elongatus* sp. PCC7942 pBG2120 exposed to ZnNPs; (**D**) *Synechococcus elongatus* sp. PCC7942 pBG2120 exposed to CuNPs. Data represent the mean ± standard deviation of at least three independent experiments.

Metallic NPs are capable to release free-ions which in most cases are involved in their toxicity processes [46]. In order to know the percentage of released free-ion from the metallic NPs, the filtrates of these NPs (see Section 2) were measured by TXRF (Table S1). In all the cases, the percentage of dissolved free-ion was less than 1.5 % showing a low amount (almost negligible) of dissolved metals from the NPs. Even so, in order to determine the released free-ions bioavailability and their possible toxic effect, the metal-detecting cyanobacterial bioreporter *Synechococcus elongatus* sp. PCC7942 pBG2120 was used. *Synechococcus elongatus* sp. PCC7942 pBG2120 is a cyanobacterial bioreporter that detects free heavy metals, which was constructed and tested previously with a range of heavy metals (Zn, Cd, Cu, Co, Hg, and Ag) [31]. In contrast to chemical methods, heavy metals bioreporters measure the bioavailable metal, which is the fraction that interacts with the organism and consequently is detected by the cells. No luminescent response was observed, indicating no free-ion bioavailability for cyanobacteria (data not shown). Nevertheless, the metal-detecting bioreporter was also tested with the metallic NPs themselves and surprisingly, a clear bioluminescent response was observed in the case of Ag, Zn and Cu NPs while TiNPs were not detected by the bioreporter (Figure 1).

As shown in Figure 1, for each metallic NP, BIF values increased in a dose-dependent fashion as a function of NP concentration (except TiNPs). The maximum BIF for ZnNPs (Figure 1C) was the highest, near 90-fold induction (after the exposure to 0.5 mg/L of NP) followed by AgNPs (after the exposure to 1 mg/L of NP) (Figure 1A) with a maximum BIF of 60-fold induction and CuNPs (Figure 1D) with a maximum BIF of 20-fold induction (after the exposure to 12.5 mg/L of NP).

### 3.4. Response of ROS-Detecting Nostoc sp. PCC7120 Bioreporters to Metallic NPs

The four ROS-detecting *Nostoc* sp. based bioreporters were tested with increasing metallic NPs concentrations.

**Figure 2:**
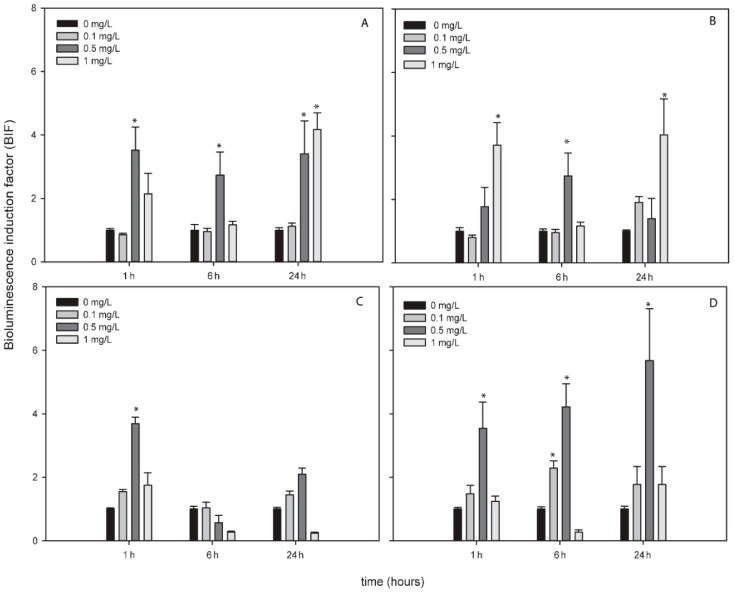
Bioluminescence induction of ROS-detecting *Nostoc* sp. PCC7120 bioreporters exposed to 0, 0.1, 0.5 and 1 mg/L of AgNPs after 1, 6 and 24 h of exposure. (**A**) *Nostoc* sp. PCC7120 pBG2154, (**B**) *Nostoc* sp. PCC7120 pBG2165, (**C**) *Nostoc* sp. PCC7120 pBG2172 and (**D**) *Nostoc* sp. PCC7120 pBG2173. The asterisks indicate statistically significant differences with respect to the control (unexposed cells) (ANOVA *p* < 0.05).

**Figure 3:**
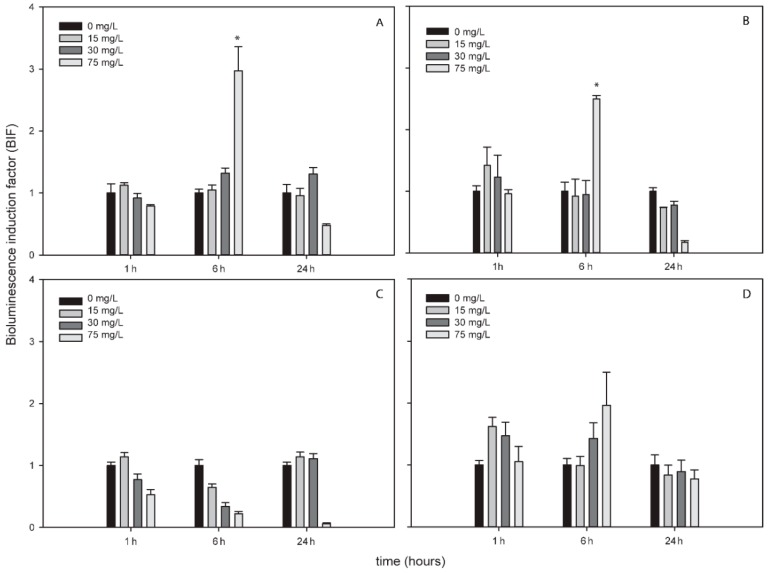
Bioluminescence induction of ROS-detecting *Nostoc* sp. PCC7120 bioreporters exposed to 0, 15, 30 and 75 mg/L of TiNPs after 1, 6 and 24 h of exposure. (**A**) *Nostoc* sp. PCC7120 pBG2154, (**B**) *Nostoc* sp. PCC7120 pBG2165, (**C**) *Nostoc* sp. PCC7120 pBG2172 and (**D**) *Nostoc* sp. PCC7120 pBG2173. The asterisks indicate statistically significant differences with respect to the control (unexposed cells) (ANOVA *p* < 0.05).

**Figure 4:**
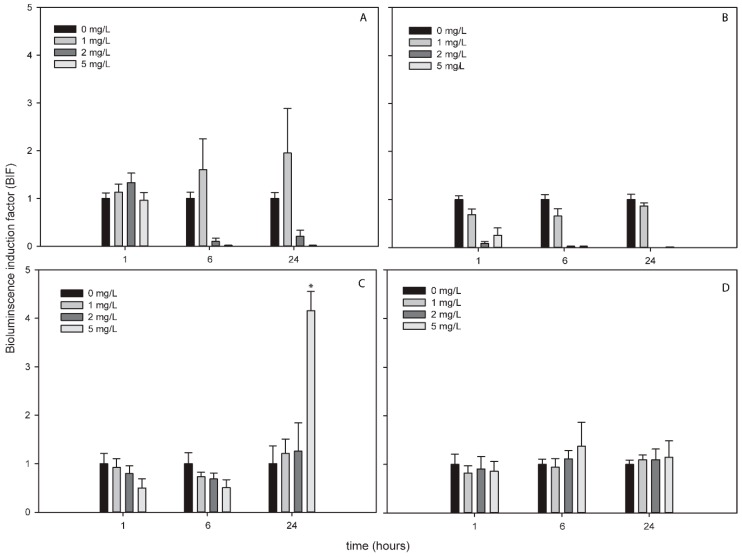
Bioluminescence induction of ROS-detecting *Nostoc* sp. PCC7120 bioreporters exposed to 0, 1, 2 and 5 mg/L of ZnNPs after 1, 6 and 24 h of exposure. (**A**) *Nostoc* sp. PCC7120 pBG2154, (**B**) *Nostoc* sp. PCC7120 pBG2165, (**C**) *Nostoc* sp. PCC7120 pBG2172 and (**D**) *Nostoc* sp. PCC7120 pBG2173. The asterisks indicate statistically significant differences with respect to the control (unexposed cells) (ANOVA *p* < 0.05).

AgNPs, TiNPs and ZnNPs induced bioluminescence in the bioreporters indicating the formation of ROS by these metallic NPs while CuNPs did not induce bioluminescence in any bioreporter and at any exposure time (Figure S2). Figure 2, Figure 3 and Figure 4 show the BIFs of the ROS-detecting bioreporters after 1, 6 and 24 h of exposure to Ag, Ti and ZnNPs, respectively. From the three metallic NPs, the high bioluminescence induction factor (BIF) was for AgNPs. The BIF was near four-fold induction with respect to the control (untreated cells) for *Nostoc* sp. PCC7120 pBG2154, *Nostoc* sp. PCC7120 pBG2165 and *Nostoc* sp. PCC7120 pBG2172 (Figure 2A–C). In *Nostoc* sp. PCC7120 pBG2172 case, this induction was observed only at shorter times of exposure (1 h) and after the exposure to 0.5 mg/L of NP; however, in *Nostoc* sp. PCC7120 pBG2154 and in *Nostoc* sp. PCC7120 pBG2165 case the bioluminescence induction was observed after 1 h and also after 24 h of exposure and after the exposure to 0.5 and 1 mg/L of NP. *Nostoc* sp. PCC7120 pBG2173 presented the maximum BIF for AgNPs, near six-fold, after 24 h of exposure and after the exposure to 0.5 mg/L of NP (Figure 2D).

TiNPs only induced bioluminescence in *Nostoc* sp. PCC7120 pBG2154 and in *Nostoc* sp. PCC7120 pBG2165 after 6 h of exposure and after the exposure to 75 mg/L of NP (Figure 3A,B). As can be seen in Figure 3, the TiNPs concentrations which induced bioluminescence were all higher than the calculated IC_50_ (15.23 ± 1.18 mg/L) because below 15 mg/L of TiNPs, no significant bioluminescence induction was observed.

The bioluminescence profile of the cyanobacterial strains after the exposure to ZnNPs was different to the other NPs since only in *Nostoc* sp. PCC7120 pBG2172 which specifically detects H_2_O_2_, statistically significant bioluminescence induction was observed (Figure 4C). This bioluminescence induction appeared after 24 h of exposure and increased approximately four-fold with respect to the control after the exposure to 5 mg/L of NP. These results showed a specifically detection of H_2_O_2_ but not superoxide anion by the ROS-detecting bioreporters after the exposure to ZnNPs suggesting that the main ROS produced by ZnNPs is H_2_O_2_.

To determine the contribution of dissolved free-ions to ROS formation, metallic NPs filtrates (which contain the released free-ions, see Table S1) experiments were performed (see Section 2). In every case, the released free-ions from metallic NPs did not induce the bioluminescence of any bioreporter (data not shown).

#### Response of ROS-Detecting *Nostoc* sp. PCC7120 Bioreporters to Metallic NPs Added to Environmental Water Samples (Spiking Experiments)

In order to know whether metallic NPs produce ROS in environmental matrices, the *Nostoc* sp. PCC7120 ROS-detecting bioreporters were exposed to the same concentrations which were used under growth medium conditions of metallic NPs but the experiments were performed spiking environmental matrices with the NPs (see Section 2). Beforehand, the bioreporters were exposed to the natural waters and no bioluminescence induction was observed (which was indicative that none of the compounds present in the waters or the physicochemical characteristics of any of them caused oxidative stress in the strains).

Of the four ROS bioreporters tested, only *Nostoc* sp. PCC7120 pBG2154 and *Nostoc* sp. PCC7120 pBG2165 responded to the NPs added in the environmental waters, and only to AgNPs. As heteroaggregation process might occur between NPs and organic matter present in the natural matrices, which usually decrease toxicity [44], this could be the reason that no bioluminescence induction was observed with the other three metallic NPs. Regarding AgNPs, as already shown in Table 2, these metallic NPs presented different size peaks in all media tested including the environmental matrices, where small sizes of around 10 nm (Glx1) and 16 nm (WWTP) were recorded that could be bioavailable to the cyanobacterial cells inducing ROs formation.

**Figure 5:**
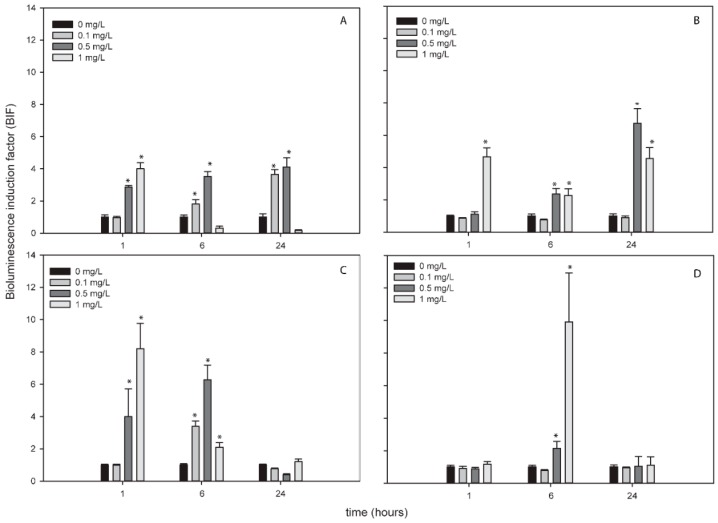
Bioluminescence response of *Nostoc* sp. PCC7120 pBG2154 (**A**,**B**) and *Nostoc* sp. PCC7120 pBG2165 (**C**,**D**) exposed to AgNPs in environmental matrices. (**A**) *Nostoc* sp. PCC7120 pBG2154 exposed to AgNPs in Glx1 water; (**B**) *Nostoc* sp. PCC7120 pBG2154 exposed to AgNPs in WWTP water; (**C**) *Nostoc* sp. PCC7120 pBG2165 exposed to AgNPs in Glx1 water; (**D**) *Nostoc* sp. PCC7120 pBG2165 exposed to AgNPs in WWTP water. The asterisks indicate statistically significant differences with respect to the control (unexposed cells) (ANOVA *p* < 0.05).

Figure 5 shows the bioluminescence response of both bioreporters to AgNPs in Glx1 and WWTP waters. *Nostoc* sp. PCC7120 pBG2154 was capable of detecting the superoxide anion produced by AgNPs in both waters (Figure 5A,B). Furthermore, in WWTP, the bioluminescence induction observed was higher, around seven-fold after the exposure to 0.5 mg/L of NP, (Figure 5B) with respect to the experiments performed in growth medium which presented a three-fold bioluminescence induction at this time and after the exposure to this AgNPs concentration.

In *Nostoc* sp. PCC7120 pBG2165 case, the bioluminescence induction in both waters after the exposure to AgNPs was around eight-fold with respect to the control (untreated cells) after the exposure to 1 mg/L of AgNPs (Figure 5C,D), higher than bioluminescence induction in growth medium (four-fold approximately).

## 4. Discussion

Due to the increased production and the widespread use of metallic NPs, their release in aquatic environments is unavoidable [47], so the study of their toxic effect in the environment is necessary. As oxidative stress is one of the mechanisms of action most observed in organisms after the exposure to metallic NPs, many studies have been performed in a variety of cell lines to describe it. To date, several studies about metallic NPs toxicity using bioluminescence bioreporters exist, however only a few of them have investigated metallic NPs ROS production [48]. Most of them have been performed with *Escherichia coli* or *Pseudomonas* based bioreporters thus, there are not any study in the literature concerning the detection of ROS produced by metallic NPs on organisms of environmental relevance such as cyanobacteria.

In this study a battery of bioluminescent cyanobacterial bioreporters to assess toxicity, bioavailable released metal free-ions and potential ROS generation by metallic NPs have been used. The applicability of this battery of cyanobacterial bioluminescent bioreporters in profiling toxicity and oxidative stress potential of metallic NPs was evaluated after the exposure to AgNPs, TiNPs, CuNPs, and ZnNPs.

General toxicity of metallic NPs was evaluated by the exposure of *Nostoc* CPB4337 to increasing metallic NP concentrations. This bioreporter strain has been previously used to assess NPs toxicity [37,38,39,49]. The calculated IC_50_ indicated ZnNPs and AgNPs as the most toxic metallic NPs tested. In general, cyanobacterial toxicity after 24 h of metallic NPs exposure was correlated with the metallic NP concentrations tested following dose-response curves.

As previously described, the toxicity caused by metallic NPs, may be induced by the NPs per se, by their released free-ions or by the combination of both [50]. For this reason, the dissolved free-ions of each metallic NP were measured by TXRF, which indicated a negligible percentage of free metal dissolved from each NP. Further information about the bioavailability of the metallic NPs and their dissolved free-ions was obtained by the heavy metal bioreporter *Synechococcus elongatus* sp. PCC7942 pBG2120. The results of these bioassays were in agreement with the values reported by TXRF, suggesting that the dissolved free-ions were available in a very low amount and not bioavailable for cyanobacteria but suggested a new application for this bioreporter as it was able to detect the metallic NPs. This new application might help to understand the bioavailability of metallic NPs in the aquatic environment.

The potential of the tested NPs to induce the production of ROS was evaluated with the ROS-inducible cyanobacterial bioreporters. One of the effects of cytotoxicity of AgNPs is related with the induction of ROS production (see review [51]) as can be confirmed by the results of ROS cyanobacterial bioreporters where after the exposure to AgNPs, the bioluminescence of all the strains was induced indicating the presence of intracellular ROS caused by AgNPs. Ivask et al. 2010 [52] used a specific *E. coli* superoxide anion luminescent recombinant strain to confirm ROS production by AgNPs, specifically superoxide anion. A further study was performed by Hwuang et al. 2008 [53] which used an *E. coli* panel of bioluminescent bacteria capable to detect different kind of ROS and they also concluded that AgNPs induced the formation of superoxide anion specifically. Although the toxicity of AgNPs has been related with their dissolved free-ions [54], no ROS formation was detected by ROS-detecting bioreporter after the exposure to Ag^+^ released free-ion suggesting that at these NPs concentration, ROS formation was produced by the NPs per se and not by the dissolved free-ions. This result confirms the results observed by Nair et al. (2013) [55] and Tlili et al. (2016) [56] where AgNPs induced more oxidative stress than their dissolved free-ions.

Although TiNPs are known as “the environmental white knight” since they are relativity harmless, inertness, and biocompatible, there exist contradictory studies about their toxicity [57]. Several studies have confirmed TiNPs ecotoxicity in aquatic environments [58,59,60]. In particular, ROS production caused by TiNPs has been previously observed in aquatic organisms [61]. After the exposure to TiNPs, only *Nostoc* sp. PCC7120 pBG2154 and *Nostoc* sp. PCC7120 pBG2165 (both harboring superoxide dismutase promoters fused to *luxCDABE*) strains responded confirming the presence of superoxide anion and superoxide dismutases implication in TiNPs detoxification. In their study, Ivask et al 2010 [52] also described the superoxide anion production after the exposure of *E. coli* based superoxide anion luminescent bioreporter to TiNPs. However, the induction of this bioreporter was observed after 7 h of exposure and at higher concentration (1000 and 3000 mg/L) of TiNPs than that of the ROS-detecting cyanobacterial bioreporters (75 mg/L). These data suggest that cyanobacterial ROS-detecting bioreporters are more sensitive than other luminescent bioreporters to detect the ROS formation after the exposure to TiNPs. Although superoxide anion was detected after the exposure to TiNPs, it was detected at higher concentrations than that of the IC_50_.At these IC_50_ values, their toxicity might be due to other mechanisms such as lipid peroxidation caused by their photocatalytic activity [62] which cannot be detected by the cyanobacterial ROS-detecting bioreporters.

The results obtained by the ROS-detecting cyanobacterial bioreporters after the exposure to ZnNPs were different to the rest of the NPs since only *Nostoc* sp. PCC7120 pBG2172 (harboring the *2-cys-prx* peroxiredoxin promoter fused to *luxCDABE* genes) was induced. This bioreporter was the only one capable to detect the H_2_O_2_ produced by the NPs suggesting that ZnNPs induced mostly the production of H_2_O_2_. Zhao et al. 2013 [63] suggested that ZnNPs had an inhibitory effect on catalase activity so H_2_O_2_ generated by superoxide dismutases was not removed completely by catalase directly and caused intracellular accumulation of H_2_O_2_. Furthermore, they concluded that GPx (gluthatione peroxidase) activity enhanced the capacity to scavenge H_2_O_2_ [63]. Another study confirmed that after the exposure of BEAS-2B cells to a sublethal concentration of ZnNPs, the expression of peroxiredoxin genes were induced [64]. In view of these results and those of the ROS-detecting cyanobacterial bioreporters, ZnNPs might generate low levels of superoxide anions that were not detected by the bioreporters but clearly generated H_2_O_2_ accumulation as detected by *Nostoc* sp. PCC7120 pBG2172. As the other metallic NPs tested, the Zn^2+^ released free-ions did not induce the bioluminescence in any ROS-detecting bioreporter, possible due to their very low concentration as confirmed by TXRF analysis.

As previously mentioned CuO NPs did not induce bioluminescence induction in any ROS-detecting cyanobacterial bioreporter. These metallic NPs have been previously reported as ROS-inducers [52,65], however in this study no ROS production was observed probably due to low ROS levels at the concentrations tested which the ROS-detecting bioreporters were not able to detect. This result might be also explained by an increase of enzymes activity to eliminate ROS in the algal cells [66]. However when the toxicity is too strong these enzymes are inhibited [67,68].

The toxicity of NPs on algae is influenced by diverse factors, specifically the characteristics and properties of the aquatic environment around algae, such as water chemistry, light and water temperature [68]. Organic matter can be adsorbed on NPs altering their surface functional groups and enhance their migration and diffusion capabilities. The coating of organic matter may limit the release of ions from metallic NPs into the water [69], prevent NP aggregation [70] and reduce the toxicity of NPs on the algae [71]. For this reason, the cyanobacterial ROS-detecting bioreporters were exposed to the metallic NPs in two spiked natural waters. Only the superoxide dismutase-based bioreporters responded to NPs exposure and only after the exposure to AgNPs. The fact that ROS production by the other metallic NPs could not be detected in the spiking experiments might have to do with heteroaggregation processes that may decrease ROS production related with the decrease in the toxicity of the NPs in real environmental samples [46]. This might not happen so clearly with AgNPs as these metallic NPs presented different size peaks in the environmental matrices, where the small sizes could be bioavailable to the cyanobacterial cells inducing ROS formation.

The data of this work confirm the use of cyanobacterial bioreporters as useful environmental tools to detect the bioavailability, toxicity and ROS production of metallic NPs. In addition, the results from the natural matrices suggest the need to validate the bioreporters with real matrices before using them in potential contaminated environments.

## 5. Conclusions

In summary, all the metallic NPs tested caused a relevant toxicity towards cyanobacteria which was dependent on the metallic NPs concentration with the following toxicity order (based on the calculated IC_50_) as ZnNPs > AgNPs > CuNPs > TiNPs. The released free-ion from each NP was found to be negligible that might account for the lack of response of the metal detecting bioreporter *Synechococcus elongatus* sp. PCC7942 pBG2120. Curiously, this bioreporter was able to detect the metallic NPs themselves, which indicate that the bioreporter might be useful to detect metallic NPs in aquatic environments. Regarding ROS formation after the exposure to metallic NPs, AgNPs and TiNPs induced the formation of superoxide anion while ZnNPs induce H_2_O_2_ formation specifically. CuNPs did not induce the bioluminescence of any ROS-detecting bioreporters suggesting no ROS formation at the concentrations tested or the formation of very low ROS levels. Finally, ROS production after the exposure to the metallic NPs in spiked natural water was observed but only for AgNPs and the results were different with respect to the growth medium conditions highlighting the importance of studying NPs impact in real environmental matrices.

## Figures and Tables

**Table 1:** Cyanobacterial strains used in this study and their culture conditions. All the reporter *luxCDABE* genes are from *Photorhabdus luminescens*.

	Gene System	Characteristics and Culture Conditions	References
*Nostoc* sp. *CPB4337*	*luxCDABE* genes in the chromosome	Toxicity bioreporter based on *Nostoc* sp. PCC7120. Nm^R^ in AA/8+N growth medium	[32]
*Synechococcus elongatus* sp. PCC7942 pBG2120	Plasmid pBG2120: *Psmt::luxCDABE* *smt* encodes the transcriptional SmtB and the metallothionein SmtA	Bioavailable heavy metal bioreporter based on *Synechococcus elongatus* sp. PCC7942 expressing the plasmid pBG2120. Cm^R^ in BG11 growth medium	[31]
*Nostoc* sp. PCC7120 pBG2154	Plasmid pBG2154; *PsodA::luxCDABE* *sodA* encodes a Mn-superoxide dismutase	Specific superoxide anion bioreporter based on *Nostoc* sp. PCC7120 expressing the plasmid pBG2154. Sp^R^ in AA/8+N growth medium	[29]
*Nostoc* sp. PCC7120 pBG2165	Plasmid pBG2165; *PsodB::luxCDABE* *sodB* encodes a Fe-superoxide dismutase enzyme	Specific superoxide anion bioreporter based on *Nostoc* sp. PCC7120 expressing the plasmid pBG2165. Sp^R^ in AA/8+N growth medium	[29]
*Nostoc* sp. PCC7120 pBG2172	Plasmid pBG2172; *P2-cys-prx:: luxCDABE* *2-cys-prx* encodes a peroxiredoxin enzyme	Superoxide anion and H_2_O_2_ bioreporter based on *Nostoc* sp. PCC 7120 expressing the plasmid pBG2172. Sp^R^ in AA/8+N growth medium	[30]
*Nostoc* sp. PCC7120 pBG2173	Plasmid pBG2173; *PkatA:: luxCDABE* *katA* encodes a Mn-catalase enzyme	Specific superoxide anion bioreporter based on *Nostoc* sp. PCC 7120 expressing the plasmid pBG2173. Sp^R^ in AA/8+N	[30]

**Table 2:** Particle hydrodynamic size determined by dynamic light scattering and ζ-potential measured by electrophoretic light scattering in ultrapure water, AA/8+N and BG11 culture media and real water matrices (Glx1 and WWTP).

Size (nm)					
	Ultrapure Water (pH 6.5)	AA/8+N (pH 7)	BG11 (pH 7.6)	Glx1 (pH 6.9)	WWTP (pH 7.5)
**Without NPs**	-	711.0 ± 181.18 171.8 ± 36.4	772.1 ± 122.6	227.9 ± 49.6	148.4 ± 10.2
**AgNPs**	55.7 ± 20.5 9.2 ± 3.3	635.9 ± 154.9 46.6 ± 10.4 8.5 ± 1.9	306.8 ± 39.1 28.96 ± 3.0	76.7 ± 38.1 10.3 ± 4.4	169.4 ± 110.0 16.5 ± 4.8
**TiNPs**	2333.0 ± 377.5	1197.0 ± 87.4	5433.0 ± 283.0	4213.0 ± 909.9	1099.0 ± 114.0
**ZnNPs**	264.8 ± 55	637.0 ± 120.3	1193.0 ± 87.5	-	509.3 ± 118.3
**CuNPs**	242.0 ± 120.2	204.6 ± 73.9	921.2±112.8	256.2 ± 91.1	249.1 ± 99.7
**ζ-potential (mV)**					
**Without NPs**	-	−27.4 ± 1.0	−11.51 ± 2.8	−9.8 ± 1.4	−9.9 ± 2.2
**AgNPs**	−10.9 ± 2.4	−24.2 ± 1.7	−21.0 ± 11.8	−4.7 ± 0.3	−11.7 ± 11.5
**TiNPs**	−17.1 ± 2.1	−23.6 ± 1.0	−18.2 ± 2.0	−17.2 ± 0.8	−12.5 ± 0.7
**ZnNPs**	−17.4 ± 0.6	−27.5 ± 1.3	−19.0 ± 2.1	−13.7 ± 3.3	−13.3 ± 1.4
**CuNPs**	−24.1 ± 0.2	−28.7 ± 0.9	−26.1 ± 1.0	−22.4 ± 0.4	−14.5 ± 0.1

-: Not detected.

**Table 3:** Inhibitory concentrations of metallic NPs that induced 10%, 50% and 90% of inhibition of bioluminescence of *Nostoc* sp. CPB4337 growth after 1, 6, and 24 h of exposure and the “R” model type fitted.

Time (h)	Metallic NPs	“R” Model Fitted	IC10 (mg/L)	IC50 (mg/L)	IC90 (mg/L)
**1**	Ag	LL.4	0.13 ± 0.04	0.23 ± 0.04	0.43 ± 0.17
	Ti	-	-	-	-
	Zn	LL2.3	-	2.04 ± 0.8	17.9 ± 5.5
	Cu	W1.3	17.01 ± 3	32.8 ± 2.1	50.01 ± 4.23
**6**	Ag	W1.3	0.02 ± 0.02	0.14 ± 0.03	1.92 ± 0.6
	Ti	W1.4	0.55 ± 0.4	8.94 ± 2.0	52.77 ± 25.0
	Zn	LL2.3	-	1.23 ± 0.1	2.96 ± 0.2
	Cu	W2.3	14.06 ± 3.2	18.58 ± 2.26	28.77 ± 2.04
**24**	Ag	W1.3	0.4 ± 0.04	0.71 ± 0.03	1.03 ± 0.05
	Ti	LL.3	3.92 ± 0.77	15.23 ± 1.18	59.2 ± 8.05
	Zn	W1.4	0.07 ± 0.04	0.38 ± 0.07	1.06 ± 0.33
	Cu	W2.3	9.17 ± 0.41	12.45 ± 0.78	20.10 ± 3.69

-: No IC value found in the tested concentration range for all NPs (0.01 mg/L to 100 mg/L).

## Supplementary Materials

The following are available online at https://www.mdpi.com/1424-8220/19/16/3597/s1, Figure S1: Dose-Response curves of the bioluminescence of *Nostoc* sp. CPB4337 after 24 h of exposure to increasing concentrations of metallic NPs, Figure S2: Bioluminescence induction of ROS-detecting *Nostoc* sp. PCC7120 bioreporters exposed to 0, 5, 15 and 20 mg/L of CuO NPs after 1, 6 and 24 h of exposure, Table S1: Dissolved free-ions concentration released from each metallic NP as calculated by TXRF after 24 h of exposure in AA/8+N medium and percentage of released free-ion metals with respect to the nominal NP concentration.
