# Supplementary material for: Use of Cyanobacterial Luminescent Bioreporters to Report on the Environmental Impact of Metallic Nanoparticles
Source: Sensors (Basel). 2019 Aug 19;19(16):3597. doi: 10.3390/s19163597 (PMC6721232; doi:10.3390/s19163597)
Supplement: Supplementary file 1 [file sensors-19-03597-s001.pdf]

sensors

MDPI

Supplementary material

Table 1. Dissolved free-ion concentration released from each metallic NP as calculated by TXRF after 24 h of exposure in AA/8+N medium and percentage of released free-ion metals with respect to the nominal NP concentration.

Metallic NP	Nominal Concentration (mg/L)	Concentration Measure by TXRF (mg/L)	% of Released Free-Ion
**AgO**	10	0.04 (Ag^{+})	0.41
**TiO_2**	100	0 (Ti^{4+})	0
**ZnO**	100	3.56 (Zn^{2+})	1.12
**CuO**	100	3.66 (Cu^{2+})	1.12

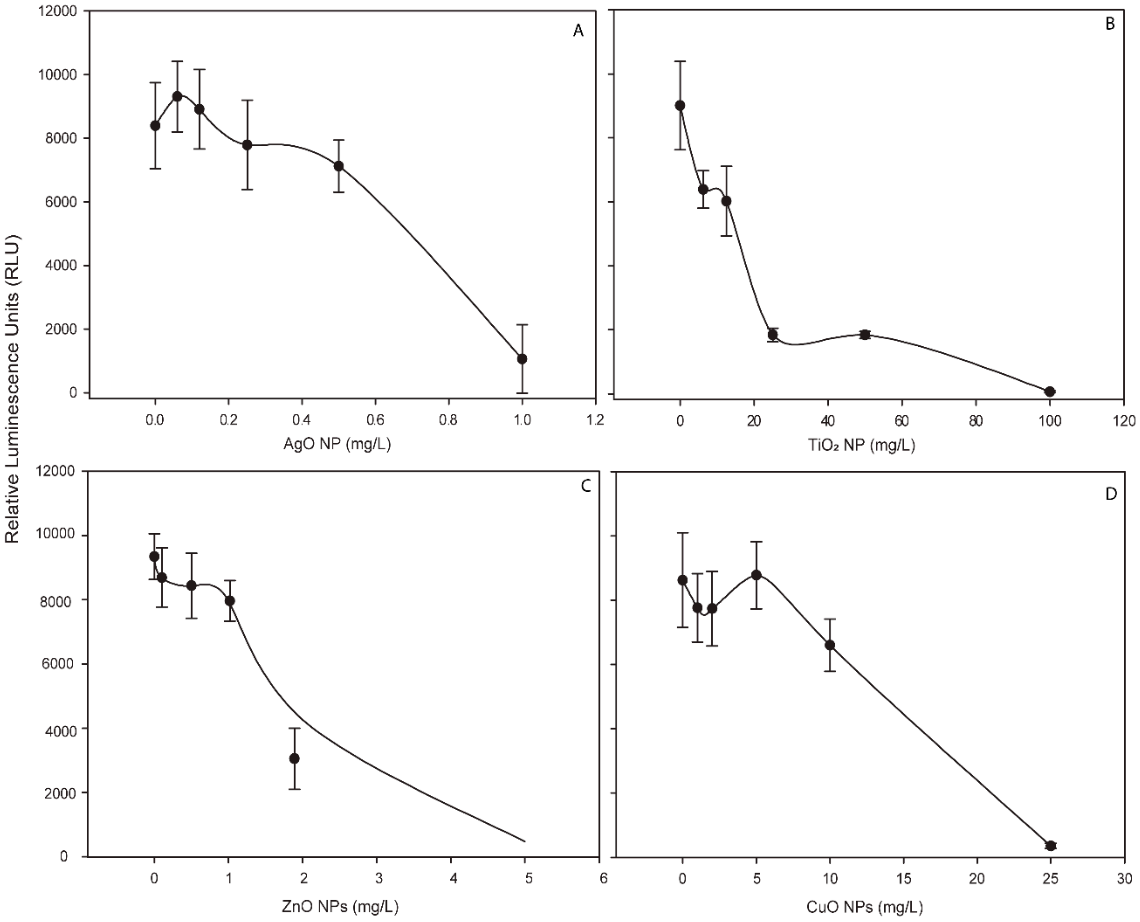

Figure S1. Dose–response curves of the bioluminescence of Nostoc sp. CPB4337 after 24 h of exposure to increasing concentrations of metallic NPs. RLU: relative luminescence units.

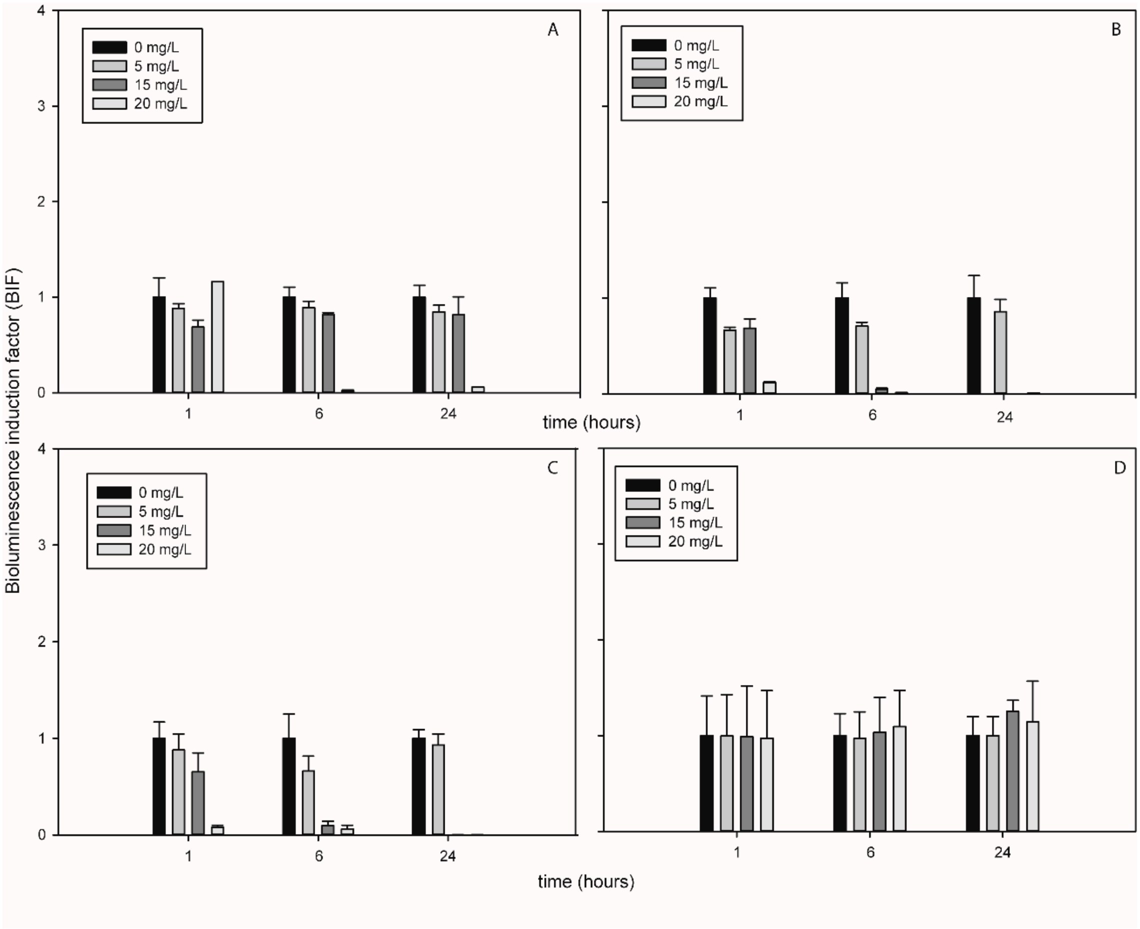

Figure S2. Bioluminescence induction of ROS-detecting *Nostoc* sp. PCC7120 bioreporters exposed to 0, 5, 15 and 20 mg/L of CuO NPs after 1, 6 and 24 h of exposure. (A) *Nostoc* sp. PCC7120 pBG2154, (B) *Nostoc* sp. PCC7120 pBG2165, (C) *Nostoc* sp. PCC7120 pBG2172 and (D) *Nostoc* sp. PCC7120 pBG2173. No significant differences ($p < 0.05$) were observed at any of the assayed times.
